# Scoping Review on the Diagnosis, Prognosis, and Treatment of Pediatric Disorders of Consciousness

**DOI:** 10.1212/WNL.0000000000207473

**Published:** 2023-08-08

**Authors:** Erika Molteni, Liane dos Santos Canas, Marie-Michèle Briand, Anna Estraneo, Carolina Colomer Font, Rita Formisano, Ekaterina Fufaeva, Olivia Gosseries, Robyn A. Howarth, Paola Lanteri, Gimena Inès Licandro, Wendy L. Magee, Vigneswaran Veeramuthu, Pamela Wilson, Tomohiro Yamaki, Beth S. Slomine, as the Special Interest Group on DoC of the International Brain Injury Association (IBIA-DoC SIG)

**Affiliations:** From the School of Biomedical Engineering & Imaging Sciences (E.M., L.S.S.C.), Faculty of Life Science & Medicine, King's College London, United Kingdom; Department of Physical Medicine and Rehabilitation (M.-M.B.), Hôpital du Sacré-Coeur de Montréal, Quebec, Canada; IRCCS Fondazione Don Gnocchi (A.E.), Florence, Sant'Angelo dei Lombardi, Italy; NEURORHB—Neuro Rehab Human Brain (C.C.F.), Fundación Hospitales Vithas, Valencia, Spain; IRCCS Santa Lucia Foundation (R.F.), Rome, Italy; Clinical and Research Institute of Emergency Pediatric Surgery and Trauma (CRIEPST) (E.F.), Moscow, Russia; Coma Science Group (O.G.), GIGA Consciousness & Centre du Cerveau2, University and University Hospital of Liège, Belgium; Department of Neuropsychology (R.A.H.), Children's Healthcare of Atlanta, GA; Neurophysiology Unit (P.L.), Fondazione IRCCS Istituto Neurologico Carlo Besta, Milan, Italy; Centro de Rehabilitación Infantil CRI CETNA (G.I.L.), Fleni, Buenos Aires, Argentina; Boyer College of Music and Dance (W.L.M.), Temple University, Philadelphia, PA; Division of Clinical Neuropsychology (V.V.), Subang Jaya Medical Center, Selangor; Division of Clinical Neuropsychology (V.V.), Thompson Hospital Kota Damanasara, Selangor, Malaysia; Department of Physical Medicine and Rehabilitation (P.W.), University of Colorado, Children's Hospital Colorado, Aurora; Division of Neurosurgery (T.Y.), Rehabilitation Center for Traumatic Apallics Chiba, National Agency for Automotive Safety and Victims' Aid, Japan; and Department of Neuropsychology (B.S.S.), Kennedy Krieger Institute; Department of Psychiatry and Behavioral Sciences (B.S.S.), Johns Hopkins University, School of Medicine, Baltimore, MD.

## Abstract

**Background and Objectives:**

Comprehensive guidelines for the diagnosis, prognosis, and treatment of disorders of consciousness (DoC) in pediatric patients have not yet been released. We aimed to summarize available evidence for DoC with >14 days duration to support the future development of guidelines for children, adolescents and young adults aged 6 months–18 years.

**Methods:**

This scoping review was reported based on Preferred Reporting Items for Systematic reviews and Meta-Analyses–extension for Scoping Reviews guidelines. A systematic search identified records from 4 databases: PubMed, Embase, Cochrane Library, and Web of Science. Abstracts received 3 blind reviews. Corresponding full-text articles rated as “in-scope” and reporting data not published in any other retained article (i.e., no double reporting) were identified and assigned to 5 thematic evaluating teams. Full-text articles were reviewed using a double-blind standardized form. Level of evidence was graded, and summative statements were generated.

**Results:**

On November 9, 2022, 2,167 documents had been identified; 132 articles were retained, of which 33 (25%) were published over the past 5 years. Overall, 2,161 individuals met the inclusion criteria; female patients were 527 of 1,554 (33.9%) cases included, whose sex was identifiable. Of 132 articles, 57 (43.2%) were single case reports and only 5 (3.8%) clinical trials; the level of evidence was prevalently low (80/132; 60.6%). Most studies included neurobehavioral measures (84/127; 66.1%) and neuroimaging (81/127; 63.8%); 59 (46.5%) were mainly related to diagnosis, 56 (44.1%) to prognosis, and 44 (34.6%) to treatment. Most frequently used neurobehavioral tools included the Coma Recovery Scale–Revised, Coma/Near-Coma Scale, Level of Cognitive Functioning Assessment Scale, and Post-Acute Level of Consciousness scale. EEG, event-related potentials, structural CT, and MRI were the most frequently used instrumental techniques. In 29/53 (54.7%) cases, DoC improvement was observed, which was associated with treatment with amantadine.

**Discussion:**

The literature on pediatric DoCs is mainly observational, and clinical details are either inconsistently presented or absent. Conclusions drawn from many studies convey insubstantial evidence and have limited validity and low potential for translation in clinical practice. Despite these limitations, our work summarizes the extant literature and constitutes a base for future guidelines related to the diagnosis, prognosis, and treatment of pediatric DoC.

## Introduction

Disorders of consciousness (DoC) including coma, vegetative state/unresponsive wakefulness syndrome (VS/UWS), and minimally conscious state (MCS) have been predominantly described in adults. Coma is a state of unresponsiveness in which the eyes are closed, and there is no arousal upon stimulation.^1^ VS/UWS is characterized by periods of wakefulness without awareness of self and/or environment.^2^ MCS includes minimal but reproducible behavioral signs of consciousness,^3^ without (MCS−) or with some evidence of language function (MCS+) (see definitions in eAppendix 1, links.lww.com/WNL/C895). DoC result from insult and disruption to brain systems that regulate arousal and awareness. A DoC for ≥28 days after brain insult or disruption is described as a prolonged DoC.^4^

In the past few years, literature has been summarized and guidelines developed for the diagnosis, prognosis, and treatment of patients with prolonged DoC. Guidelines were developed by groups in the United States,^4^ United Kingdom,^5^ and Europe.^6^ These guidelines provide little support for the evaluation and management of pediatric DoC, for whom dedicated literature is lacking overall. The European and UK guidelines were based entirely on adults. While Giacino and colleagues included pediatric literature in their systematic review,^4^ only 3/18 recommendations focused on children. One provides indications for clinical practice, and 2 recommend counselling to families on the lack of prognostic and therapeutic evidence.

Studying children with DoC presents multiple challenges due to small sample sizes at single sites and heterogeneity in age and etiology of injury. Assessment of young children with DoC is difficult due to immature nervous systems and limited repertoire of developmental skills.^7,8^ Although existing literature on DoC has focused primarily on adults, in 2022, a systematic search^9^ on neuroimaging and neurophysiologic methods for the diagnosis and prognosis of children with DoC found preliminary evidence for the application of event-related potentials to support diagnosis. A commentary also reviewed treatments available to this population.^10^

In this context, a working group of expert members of the Special Interest Group on DoC of the International Brain Injury Association^11^ conducted a scoping review on the diagnosis, outcome/prognosis, and treatment of pediatric DoC. The aim was to identify and summarize existing literature on diagnosis, assessment tools, prognostic factors, and treatment approaches for pediatric DoC. Analyzing the scope of this literature is a preliminary step in the development of evidence-based guidelines on the evaluation and management of pediatric DoC.

## Methods

### Primary and Secondary Outcomes

Primary outcomes include systematic identification and summary of published evidence describing diagnostic and outcome/prognostic methods of assessment and therapeutic management of children with DoC and generation of summative statements. We considered DoC lasting for ≥14 days (rather than 28 days^4^) to maximize inclusion of available cases and cohorts, given the paucity of literature on pediatric DoC.^12^ We considered peer-reviewed published documents including novel data describing children and adolescents aged between 6 months and 18 years at onset of a neurologic injury or insult resulting in DoC. The Secondary outcome is the identification and description of levels of evidence associated with reported results in selected documents. This scoping review was reported in accordance with Preferred Reporting Items for Systematic reviews and Meta-Analyses–Extension for Scoping Reviews guidelines.^12^

### Eligibility Criteria

Full-text articles had to include novel data from children, adolescents and young adults (1) aged 6 months through 18 years at onset of DoC, (2) with DoC (including terms such as coma, VS/UWS, MCS, emergence from MCS [eMCS] or Glasgow Coma Scale [GCS] score ≤8), (3) with DoC duration ≥14 days, and (4) with non-neurodegenerative conditions.

### Search Strategy and Selection Criteria

**Figure 1:**
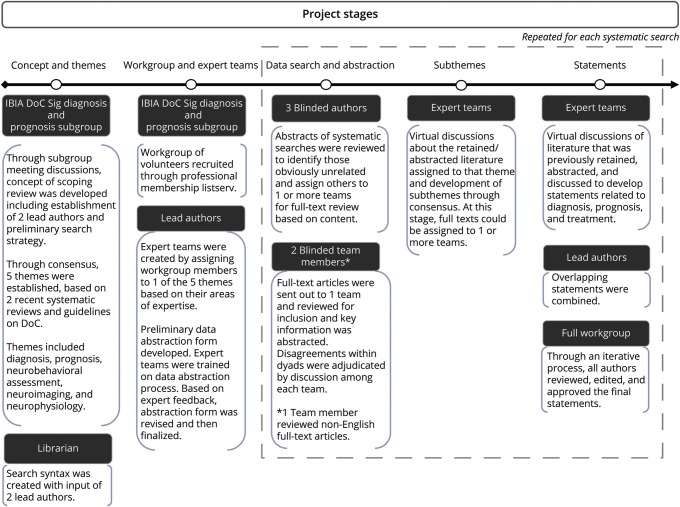
Workflow Illustrating the Generation of Themes, Subthemes, and Statements Through the Study The IBIA DoC-SIG Diagnosis and Prognosis Subgroup conceptualized the study and established 5 themes a priori, based on the previous work and recommendations for the general population with DoC and based on the terms included into the database search. Three systematic searches were repeated during the study, with 4 different databases interrogated at any search. Subthemes were iteratively generated, which informed the statements formulation. DoC = disorder of consciousness; IBIA-DoC SIG = Special interest Group on Disorders of Consciousness of the International Brain Injury.

To identify potentially relevant studies, 4 equivalent logic searches were performed in 4 databases (PubMed, Embase, the Cochrane Library, and Web of Science) on September 28, 2019 (Sintax is available in eAppendix 2, links.lww.com/WNL/C895). Searches included terms related to DoC (e.g., VS), age (e.g., child, adolescent), and assessment tool or treatment (e.g., EEG). They comprised terms disused in the field (e.g., apallic state). We considered the logic union ∪ (i.e., all documents in unique copy) of the studies. Relevant references from previous recommendations for DoC in adults^4^ were double-checked for quality control. Two additional identical searches were repeated in the 4 databases on August 15, 2021 (manuscript preparation) and on November 9, 2022 (manuscript revision) to identify recent studies (Figure 1).

### Data Identification, Screening, and Eligibility Verification

Three blind abstract reviewers identified in-scope documents by verification of eligibility criteria. Full-text articles were assigned to 1 of 5 thematic teams. Themes were identified a priori based on previous independent work^6,13^ and logic search terms: diagnosis, prognosis, neurobehavioral techniques, neuroimaging, and neurophysiology techniques. Team members received preliminary training on inclusion/exclusion criteria, data collection process, and double-blind procedure before starting the review process (Methods details are available in eAppendix 3, links.lww.com/WNL/C895). Each full-text article was reviewed double-blind using a standardized abstraction form. Disagreements were resolved conservatively by consensus. For selected studies (i.e., eligible studies satisfying inclusion criteria), data were collected double-blind including the study design and aim, patients' demographic information at group/subgroup/individual level, diagnosis, clinical assessment and support needed, treatment, behavioral, imaging, neurophysiologic, and biological examinations, conclusions of the study, sources of bias, and evidence (eAppendix 4). For relevant articles, Quality Assessment of Diagnostic Accuracy Studies 2 (QUADAS-2)^14^ and Prediction Model Risk of Bias Assessment Tool (PROBAST)^15,16^ checklists were completed. Full-text articles written in Italian, French, Portuguese, Japanese, Russian, and Spanish were reviewed by 1 expert only. Each informative document was graded per level of evidence according to Cochrane framework and associated with high/medium/low evidence based on abstraction form questions Q67-Q82, QUADAS-2, and PROBAST (Results available at doi.org/10.10.5281/zenodo.7997317).

Depending on thematic contents of each article, documents were reevaluated by 1 or more teams. Teams were instructed to examine the aim and main topic of discussion of each article, to identify similarities between articles, and to cluster articles into subthemes (including any subthemes related to treatment). At least 1 team discussed each retained article, highlighted main observations and conclusions, and produced a critical summary of articles within each subtheme. Next, each team generated summative statements of clustered literature (eAppendix 3, links.lww.com/WNL/C895; doi.org/10.10.5281/zenodo.7997317); these were subsequently merged by the lead authors and iteratively reviewed by all authors until consensus. A set of data elements to be reported in pediatric DoC research was identified through data mining and item discussion until agreement. Detailed description of the search, screening process, data extraction, assignment of articles to themes and subthemes, and framework for the generation of summative statements can be found in eAppendices 3 and 4.

### Level of Evidence

For each summative statement, highest level of single-study supporting evidence was rated as high (+++), if supported by > 1 controlled clinical trial; medium (++), by > 1 robust well-designed cohort/group study; and low (+), by case studies, series, or studies with a high risk of bias only (Adapted from Reference 17, Tables 1–3). Number of supporting documents and an evidence score were calculated.

**Table 1:**
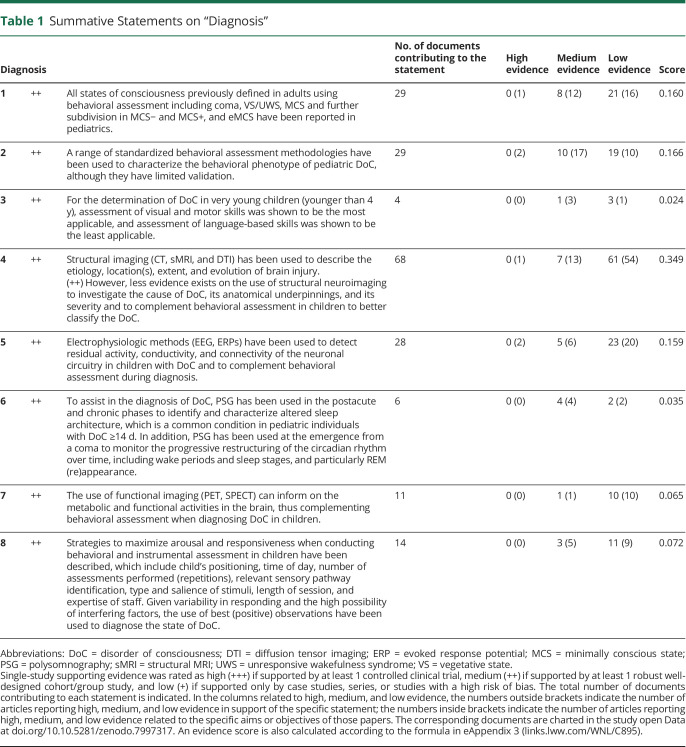
Summative Statements on “Diagnosis”

**Table 2:**
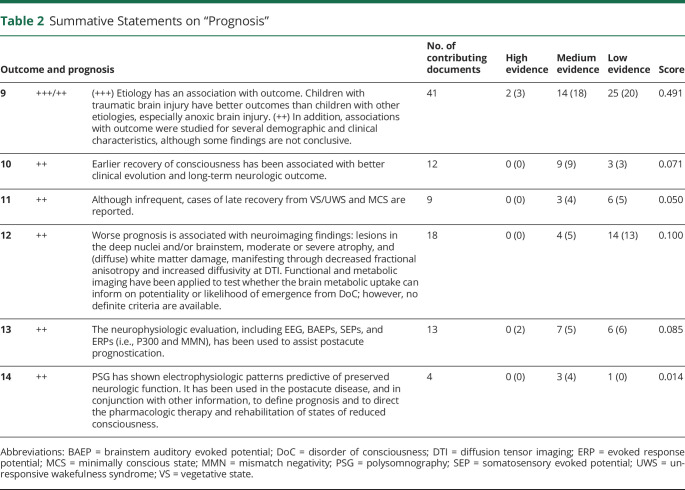
Summative Statements on “Prognosis”

**Table 3:**
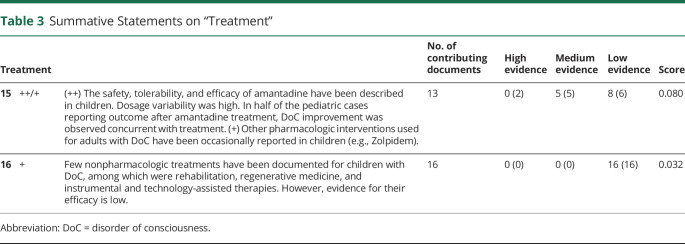
Summative Statements on “Treatment”

### Standard Protocol Approvals, Registrations, and Patient Consents

Ethical approval was not required because of the nature of the study (no humans or animals).

### Data Availability

Data generated in this study are provided as supplemental information. According to UK research councils' Common Principles on Data Policy, and according to Wellcome Trust's Policy on data, software, and material management and sharing, all data supporting this study are openly available at https://zenodo.org/record/7997317.

## Results

### Literature Identification and Selection

**Figure 2:**
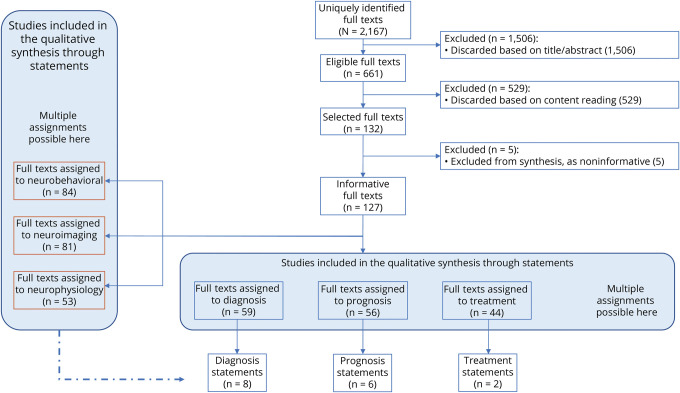
Flowchart Illustrating the Extraction of Evidence on the Diagnosis, Prognosis, Treatment, and Techniques Used in the Care of Pediatric Patients With DoC The selected full texts (n = 132, of which n = 127 informative) were in most cases reviewed by more than 1 team of experts, as relevant to more than 1 topic. Full texts were reviewed for information about clinical aim (diagnosis, prognosis, and/or treatment) and for the techniques used (behavioral tests, neuroimaging, and neurophysiologic examination). DoC = disorder of consciousness.

Initial search retrieved 776 results from PubMed; 506 from EMBASE; 84 from Cochrane; and 471 from Web of Science. After removal of duplicates within and between searches, 1,486 abstracts were screened. The second search retrieved 102 additional documents, and the third retrieved 579. Overall, the screening phase identified 2,167 documents (Figure 2; More details are available in eAppendix 5, links.lww.com/WNL/C895). Articles written in German (n = 28), Chinese (n = 22), Polish (n = 7), Czech (n = 3), Croatian, Dutch, Lithuanian, Romanian, and Serbian (n = 1 each) were discarded in the screening phase due to lack of language support by the working group. After blinded review, 132 articles were judged eligible to be retained. The selected group included 1 article in French, 5 in Japanese, 2 in Portuguese, and 2 in Russian.

### Literature Composition

The list of retained articles is provided in eAppendix 6 (links.lww.com/WNL/C895). Of the 132 retained documents, 25% were published in the past 5 years (33/132) (Details are available in eAppendices 7 and 8). Overall, 2,161 individuals met inclusion criteria (eAppendix 7); 33.9% were female patients (527/1,554). Of 118 articles clearly reporting cohort age, 19 (16.1%) were on infants and/or toddlers (older than 6 months; aged 3 years or younger); 67 (56.8%) children (older than 3 years; aged 12 years or younger), and 32 (27.1%) adolescents (older than 12 years). Etiologies included traumatic brain injury (TBI, 40/132; 30.3%), hypoxic/anoxic brain injury (19; 14.4%), stroke (5; 3.8%), encephalitis of infectious and other causes (20; 15.2%), mixed (36; 27.3%), and other/unclear (12; 9.0%). Fifty-seven (43.2%) were single case reports, and 21.2% reported results from more than 20 cases. Observational studies were 56 (42.4%), and clinical trials were 5 (3.8%). Level of evidence was high in 5 (3.8%), medium in 47 (35.6%), and low in 80 (60.6%) (eAppendix 9).

Five articles were judged noninformative during qualitative synthesis because conclusions were unrelated to DoC, or based on cohorts that included cases <14 days. Of the remaining 127 articles, 84 (66.1%) included neurobehavioral measures, 81 (63.8%) included neuroimaging, and 53 (41.7%) neurophysiologic measures. Fifty-nine (46.5%) were related to diagnosis, 56 (44.1%) to prognosis, and 44 to treatment (34.6%) (eAppendix 10, links.lww.com/WNL/C895). Qualitative review by expert teams revealed 6–10 subthemes within each theme (eAppendices 11–16).

### Subtheme Identification

Seven diagnosis subthemes were identified (eAppendix 11, links.lww.com/WNL/C895). The largest included articles on multimodal diagnostic approaches that used neurobehavioral assessment combined with neuroimaging and/or neurophysiologic methods. Ten prognosis subthemes were identified (eAppendix 12). The largest 3 investigated etiology, clinical factors, and neurophysiology as predictors of outcomes. The largest treatment subtheme was pharmacology (eAppendix 13).

### Main Evidence

Neurobehavioral tools used most frequently included the Coma Recovery Scale–Revised (CRS-R), Coma/Near-Coma Scale (CNCS), Level of Cognitive Functioning Assessment Scale (LOCFAS), and Post-Acute Level of Consciousness Scale. Among imaging techniques, structural CT and MRI were reported most frequently, although some studies described the use of PET, functional MRI, and/or MR spectroscopy. The neurophysiologic technique used most frequently was EEG. Other methods used included event-related potentials (e.g., evoked response potentials [ERPs], somatosensory evoked potentials [SEPs], brainstem auditory evoked potentials [BAEPs]) and polysomnography (PSG) (eAppendices 14–16, links.lww.com/WNL/C895).

Sixteen statements were created, including 8 for diagnosis, 6 for prognosis, and 2 for treatment (Tables 1–3). To increase the level of evidence in future research on pediatric cases with DoC, a set of data elements were identified, which could guide reporting (Table 4).

**Table 4:**
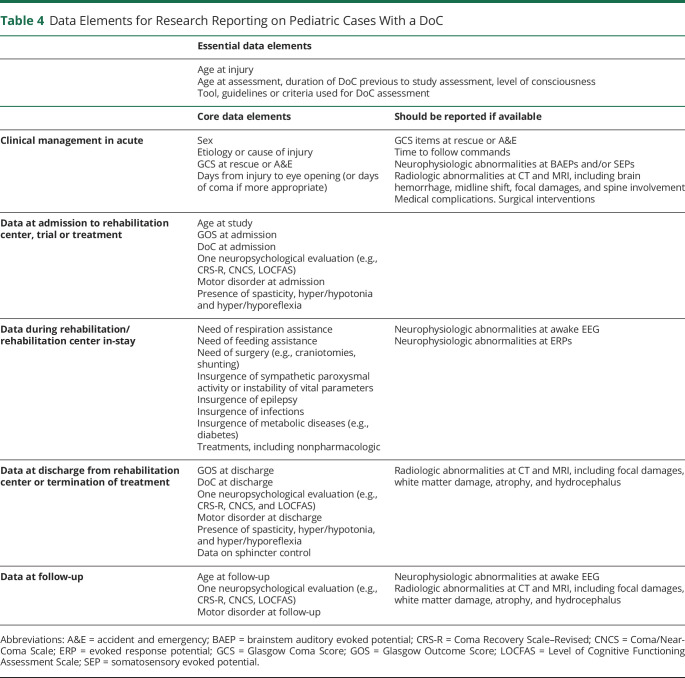
Data Elements for Research Reporting on Pediatric Cases With a DoC

### Diagnosis

#### Statement 1. (++) Definitions of States of Consciousness

##### Evidence

(++) Literature investigating children and adolescents with DoC used definitions of coma, VS/UWS, MCS, and eMCS formulated for adults. Coma is a DoC by the European guidelines^6^ but is excluded from DoC spectrum by other recommendations.^3,4^ The classification of VS/UWS and MCS (including MCS− and MCS+) was reported in children older than 6 years.^18,19^ Some evidence^7^ also exists in younger children (mean age = 2.7 years). Further research is needed to explore the utility and suitability of these terms for the youngest children and those with preexisting neurodevelopmental disabilities.

#### Statement 2. (++) Behavioral Assessment

##### Evidence

(++) The CRS-R was used most frequently. It was used in several single case studies, larger studies,^20, 21^ and combined with instrumental measurements.^22^ Only 1 study explored the psychometric properties of the CRS-R in children.^23^

Measures with some validation in pediatric DoC include the LOCFAS,^24-26^ western neurosensory stimulation profile,^27^ and CNCS.^21,28,29^ Qualitative analysis of behavior was used to characterize DoC in children^7,19^ based on accepted characteristics of DoC stages.

#### Statement 3. (++) Very Young Children (Younger Than 4 Years)

##### Evidence

(++) DoC states were identified in children younger than 4 years.^7^ Language skills develop rapidly over early childhood in typically developing children. In very young children believed to be in MCS, visual fixation and pursuit are commonly observed, while intelligible verbalizations and command following occur rarely.^7,29^ Age-appropriate salience, familiarity, and emotional relevance of stimuli have also been considered. When using neurobehavioral assessment tools designed and validated on adult cohorts with very young children, some children in MCS may be inaccurately classified due to limited language development and sensorimotor limitations.^7^

#### Statement 4. (++) Structural Imaging

##### Evidence

We define structural imaging as all instrumental approaches, specialized for visualization, qualitative, and quantitative analyses of anatomical properties of the brain, including their deviation from a normative (e.g., atrophy, anatomical lesions, etc). Acutely, structural neuroimaging was widely used to identify etiology, perform differential diagnosis, and investigate brain injury evolution^30^; and in combination with neurophysiologic examination.^31^ (++) Few studies used neuroimaging to formulate a DoC diagnosis or to characterize DoC of ≥14 days. After traumatic DoC diagnosis, structural neuroimaging was used to describe (partial) preservation of structures, pathophysiology, and brain areas where recovery of function can be considered.^32^ In research, structural neuroimaging was shown to assist in identifying neuroanatomical correlates of pediatric DoC at group level, including time-evolving relation between secondary neural damage and behavioral changes.^33^

#### Statement 5. (++) EEG and Evoked Potentials

##### Evidence

(++) In children with VS/UWS, EEGs were used to confirm consciousness status when wide cortical damage presented with brain stem preservation^34^ to detect residual cortical activity and connectivity, revealing potential for improvement and to study common DoC complications, such as epileptic abnormalities.^35^

Stimulation paradigms combined with ERP recordings were used to assess responsiveness in children with DoC.^22^ Among these, auditory oddball (presentation of sequential repetitive stimuli, infrequently interrupted by a deviant stimulus) provided an alternative to language-based stimulation when there was doubt on the individual's capability to access instructions, commands, or language content. In general, a stimulus that is salient, emotionally relevant, and familiar increases likelihood to generate cortical electrophysiologic response.^31^ Semantic paradigms combined with ERPs assess conscious processing of words. P300 component was observed in healthy controls, and in approximately 50% of cases with MCS, but not in pediatric VS/UWS.^22^ Presence of P300, and later mismatch negativity (MMN) component, is regarded as an indicator of high consciousness level and as predictive of favorable outcome.

Among ERPs, SEPs have been proposed as reliable instrumental biomarkers in postacute pediatric DoC, although they might not be observable nor repeatable in the entirety of healthy children.^18,22^ ERP paradigms in pediatric DoC are limited by frequent movement artifacts, which sometimes require correction. The large number of repetitions necessary to obtain a reliable trace of neural responses can occasionally cause unacceptably long acquisition times for examination due to the patients' quick fatigability, short arousal, and frequent attention fluctuations. Sensory deficits caused by damage to sensory pathways can be an additional impeding factor.^22^

#### Statement 6. (++) PSG

##### Evidence

(++) DoC are associated with severe alterations in sleep duration and architecture and decreased complexity of the polysomnographic pattern.^36^ PSG was proposed to assist in the differential diagnosis of VS/UWS from hypothalamic dysfunction such as hypothalamic-induced lethargy,^20,36^ to identify temporal slow wave activity (SWA) profiles^20^ and the (re)appearance of sleep spindles,^36^ and to document transitions between DoC levels.

PSG can identify REM sleep presence, which is considered sign that conscious experience (i.e., lucid dreams) is possible.^36^ Sleep architectures are not always representative of behavioral diagnoses, and misclassifications are estimated at 22%.^20^ Fluctuation between VS/UWS and MCS can be potential confounding variable; in addition, medications (e.g., antiseizure) can alter sleep pattern and microstructure.^36^ Although some sleep features might be concealed or distorted due to medications,^36^ PSG monitoring is proposed for assessing the ability of the brain to stabilize in specific stages, to switch between stages,^36^ and to generate figures and microstructures indicative of underlying integrity of specific circuits.^20^ At emergence from coma, this ability can evolve quickly, and it was shown to anticipate the corresponding behavioral evidence in some cases.^37^

Scoring of PSG of children with DoC can be qualitatively performed through the Sleep Patterns for Pediatric UWS tool.^36^

No studies reported on PSG use in intensive care units (ICUs) in pediatric DoC, probably due to technique unwieldiness, need of time-consuming expert visual review, and interference of pharmacologic sedation and ICU environmental factors.

#### Statement 7. (++) Functional Imaging

##### Evidence

(++) Severe brain hypoperfusion at PET and SPECT is a marker of decreased arousal and responsiveness in postacute and chronic phases.^38^ VS/UWS in children is characterized by generalized (chronic) decreased metabolic activity in the brain, including reduction in glucose uptake at PET.^39^

In children with unclear DoC states, 18F-fluorodeoxyglucose (FDG)–PET was used to demonstrate persistent global reduction of cerebral glucose metabolism as evidence of VS/UWS.^39^ However, there is no sufficient evidence that FDG-PET alone can be used to reliably confirm a clinical diagnosis of VS/UWS in children in postacute or chronic phases.

In addition, FDG-PET and SPECT were proposed to assist monitoring of brain metabolism after pioneering treatments for pediatric DoC,^35^ to detect possible functional changes or recovery. No evidence was found for use of near-infrared spectroscopy in pediatric DoC, possibly due to several factors, including general inability of photons to reach the deep structures of the brain (>4 cm underneath the scalp) in individuals other than newborns, risk of invalid measures due to hydrocephalus, and limited validity of current homogeneous spherical models of brain tissue in the presence of focal anatomical lesions.

#### Statement 8. (++) Maximization of Arousal and Responsiveness

##### Evidence

(++) Literature has pointed out the need of robust and reliable stimulation to maximize arousal, including stimulation saliency^40^ attending to the child's position, choosing optimal times of day for assessment,^41^ employing trained and experienced staff in structured assessment scoring,^27^ and using serial assessment (≥3) to characterize DoC.^24,41^ Using only the best (positive) observations has implications in cautious and optimistic determination of prognosis.^22^

Delivery of long assessment sessions has been reported to induce patient fatigue, resulting in false-negative results, which are not reflective of inability to respond, but rather related to the reversible patient's exhaustion.^22^ Tasks based on different modalities (i.e., visual, auditory, and tactile) have been used to overcome potential damages to specific sensory pathways and maximize the chance to instrumentally observe neural activation, which in some circumstances is indicative of consciousness.^18,22^

### Outcome and Prognosis

#### Statement 9. (+++/++) Modulators of Outcome

##### Evidence

(+++) Several cohort studies (n = 127,^42^ n = 86,^24^ and others) and smaller case series compared outcomes of children with TBI with those with other etiologies (anoxic injury only or mixed etiology including anoxia, infection, status epilepticus, and stroke). Children with TBI had better outcomes than those with other etiologies. Anoxic brain injury has been associated with worse outcomes. Two studies included a common subgroup of 26 individuals.

(++) Among clinical predictors of prognosis, literature investigated effects of age,^26,28,43^ location and extent of brain lesions, presence of epilepsy, and instability of vital parameters on neurologic outcome. Findings were either not conclusive or contradictory.

#### Statement 10. (++) Early Recovery

##### Evidence

(++) One cohort study^43^ (n = 56) and 1 case series (n = 3) of children with anoxic injury showed that a shorter length of DoC (60–90 days) was associated with a better outcome. Other cohort studies showed that early neurobehavioral assessments (<3 months) were related to long-term state of consciousness (at 6 months; n = 92)^29^ and predictive of DoC 5 years postinjury (n = 124).^26^

Several predictors of prognosis were reported: time to follow commands (i.e., days from injury until patients follow commands or until a GCS motor score of 6),^44^ responsiveness at admission,^28^ and social and motor responsiveness.^43^ Among 11 behavioral predictors, response to stimuli, execution of commands, and evidence of awareness of self <3 months after injury were most strongly linked to a positive prognosis.^26^

#### Statement 11. (++) Late Recovery

##### Evidence

(++) Rare cases of late emergence from VS/UWS or MCS are reported >12 months from TBI and >6 months from non-TBI. One study^45^ showed that 4/27 patients with DoC for ≥90 days developed consistent verbal communication 3–14 years after injury. Six additional case reports^33^ documented late recovery from DoC (9 months–7 years). All 10 cases were older than 13 years at injury, with TBI etiology in 7/10 cases. Another study showed that a minority of individuals with DoC manifested significant functional recovery between 1 year after injury and later follow-up (2–12 years).^19^ Among factors proposed to favor recovery from DoC >1 year were older age, TBI etiology, appropriate nursing care, protracted intensive rehabilitation, successful seizure management, and complication resolution (e.g., infections, cranioplasty).

#### Statement 12. (++) Neuroimaging

##### Evidence

(++) In children with DoC >14 days, structural imaging was used to assess brain atrophy, deep nuclei lesions, and secondary white matter damage and to predict neurologic deficit.^31^ In pediatric VS/UWS and MCS, severity of clinical disability correlates with white matter tract abnormalities, reduced fractional anisotropy, and increased diffusivity in corpus callosum and superior cerebellar peduncles.^46^ Tracking of white matter tracts using diffusion tensor imaging presents technical challenges in children younger than 24 months and needs to be methodologically addressed.^46^

Chronic brain hypoperfusion after injury was found to relate to outcome, with severe hypoperfusion interpreted as lack of sufficient metabolic support to enable emergence from VS/UWS.^38,39^ While some pediatric normative data are available, identification of intraindividual standard reference regions for fully quantitative PET computation is still debated.

#### Statement 13. (++) EEG and Evoked Potentials

##### Evidence

(++) BAEP and SEP abnormalities are indicators of brainstem and/or encephalic damage and point at poor prognosis, especially in anoxic children.^47^ Although used acutely, ERPs are less common but potentially equally useful to establish outcome in postacute settings (≥14 days). Absence of cortical component N20 on bilateral SEPs was observed in relation to wide cortical lesions with preserved N13 brainstem component and generally poor outcome at 6 months,^47^ although a few comatose children showed progression to mild or moderate neurologic deficits.^48^ Absence of MMN from auditory evoked potentials was also considered an unfavorable feature in VS/UWS and especially in MCS. Combined use of EEG and evoked potentials was seen to improve the outcome prediction in comparison with the use of just 1 modality.^47^

#### Statement 14. (++) PSG

##### Evidence

(++) Reappearance of sleep spindles and REM sleep after coma indicates integrity of supporting neural circuits and increases likelihood of transition from VS/UWS to the MCS and eMCS.^36,37^

Differential EEG activity during sleep and wake was observed to be related to later recovery from pediatric VS/UWS,^47^ particularly in the beta band,^37^ and to anticipate later neurobehavioral scores.^37^ Reduction in parietal SWA buildup was more frequent in individuals with DoC and lowest in those with poorest outcome.^20^ From early childhood to late adolescence, the location of maximal SWA undergoes a shift from posterior toward anterior brain regions.^20^

Several factors hamper prognosis using PSG. Epilepsy and pharmaceuticals can distort sleep macrostructure and microstructure, and spasticity can cause multiple awakenings during the night.^20^

### Treatment

#### Statement 15. (++/+) Pharmacology

##### Evidence

(++) Dopamine agonists were used to promote recovery of function in the mesocircuit after pediatric DoCs and their administration was reported in 9 articles (2 small clinical trials; 2 small cohort studies^49,50^). Overall, amantadine was administered to 53 children; 6 also received pramipexole and 2 methylphenidate. In 29/53 (54.7%) cases, DoC improvement was observed after treatment; in 9/53 (17.0%), improvement was unclear. Neurobiological functions supporting amantadine effectiveness are still partially unknown.

(+) Zolpidem, a pharmaceutical with γ-aminobutyric acid effect, was administered to 6 children, in 3 independent studies. Only 1/6 had some DoC improvement during treatment, mainly attributed to an associated immunotherapy, 2 had unclear benefit, and 3 had no observable benefit from zolpidem. Zolpidem was also tested as adjunctive treatment to accelerate recovery and rehabilitation results in a case of anti-NMDA receptor encephalitis after tumor removal and immunotherapy, with clinically significant response.^51^

#### Statement 16. (+) Nonpharmacologic Interventions

##### Evidence

(+) In 1 series and 1 single case, benefits of multisensory stimulation and utility of long delayed intensive rehabilitation programs were discussed for slow-to-recover children with DoC and in relation to neuromotor and neurocognitive domains. Regenerative treatments using cell transplantation were reported for 9 patients. Assistive technologies to improve communication in home environment, median nerve stimulation, and traditional medicine therapies were also tested on single cases (eAppendix 13, links.lww.com/WNL/C895).

## Discussion

This review systematically examines all evidence published before November 9, 2022, on pediatric DoC. It relies on definitions of coma, VS/UWS, MCS, and emergence from MCS previously adopted^3,4^ (more details available in eAppendix 1, links.lww.com/WNL/C895). Minimal duration of DoC is of 14 days from severe brain injury and prognosis is an outcome prediction thereafter. Strengths include 7-language systematic literature review, by experts from 10 countries, and diverse specialties. Literature was examined to find potential bias. One hundred thirty-two articles were pooled and evidence was described into summative statements to inform the clinics. An increase in published studies was noted over the recent years. Most retained reports were single cases, series, or small observational studies. Studies with stronger designs mostly provided a single case or small subset that met criteria for inclusion. Strength of evidence was low, and it was not possible to draw conclusions from many individual studies retained. Despite this, systematic pooling of results enabled identification of subthemes and replicated findings. Several statements related to diagnosis, outcomes/prognosis, and treatment were identified to potentially focus future research endeavors. Statements might be used along with expert opinion to create guidelines and inform practice.

Inclusion and exclusion criteria were applied consistently throughout the article evaluation and across teams; however, at times, interpretation was challenging. We included cases with DoC lasting ≥14 days, differently from current recommendations for the general population with DoC, requiring length ≥28 days^4^; this pragmatic choice maximized inclusion and evidence, yet resulted in few uncertain cases for whom DoC duration could not be clearly extracted, and in differences between teams, with possible overinclusion. Information about time when individuals transitioned from coma to UWS was rarely reported. Sedative administration was not reported in most cases, which caused uncertainty around the exclusion of cases for whom coma was pharmacologically induced. In addition, while we included children aged 6 months and older, our inclusion criteria did not specify whether this was age at event or at study, which also was interpreted differently across teams. That said, few studies investigated neonates and infants younger than 6 months. For large studies having a subgroup of children with DoC, demographic and clinical information was only extracted for the relevant subgroup; however, demographics were not always reported for subgroups, which were often small or even single cases, and in few cases, we could not rely on the general conclusions driven from the entire cohort. Last, despite being systematic, this review is not a single-patient meta-analysis. Large cohort studies did not provide sufficient details to conduct analyses at individual level, which would introduce further bias toward low evidence case studies and series or would require correspondence with the authors.

To increase the level of evidence, a minimal information set should be reported in future scientific literature: (1) age at event, (2) age at study, (3) GCS at event, (4) etiology, (5) at least 2 assessments of state of consciousness, ideally using a standardized behavioral measure (e.g., CRS-R) and possibly at admission and discharge from rehabilitation, (6) occurrence or absence of (a) epilepsy, (b) craniotomy/decompressive surgery, and (c) paroxysmal activity, (7) medications, and (8) rehabilitation received (Table 4). Negative findings should be reported. Whenever possible, length of DoC should be documented, and children with DoC ≥14 days should be analyzed separately from those with a shorter DoC. Last, if appropriate, a measure of global functioning (such as the Functional Independence Measure for Children and/or the Disability Rating Scale) should be reported.

Literature on pediatric DoC is mainly observational, and clinical details are either inconsistently presented or absent. Conclusions drawn from many studies convey insubstantial evidence, have limited validity, and low potential for translation in clinical practice. Despite these limitations, several statements related to diagnosis, outcomes/prognosis, and treatment were identified that can inform future research. Together with expert opinion, our work constitutes a base for future guidelines on the management of pediatric DoC.

## Glossary

BAEP: brainstem auditory evoked potential
CNCS: Coma/Near-Coma Scale
CRS-R: Coma Recovery Scale–Revised
DoC: disorder of consciousness
eMCS: emerged from MCS
ERP: evoked response potential
FDG: fluorodeoxyglucose
GCS: Glasgow Coma Scale
ICU: intensive care unit
LOCFAS: Level of Cognitive Functioning Assessment Scale
MCS: minimally conscious state
MCS−: minimally conscious state minus (i.e., with language comprehension and production deficit)
MCS+: minimally conscious state plus (i.e., with some language abilities)
MMN: mismatch negativity
PROBAST: Prediction Model Risk of Bias Assessment Tool
PSG: polysomnography
QUADAS-2: Quality Assessment of Diagnostic Accuracy Studies 2
SEP: somatosensory evoked potential
SWA: slow wave activity
TBI: traumatic brain injury
UWS: unresponsive wakefulness syndrome
VS: vegetative state
